# Examining Health and Wealth Correlates of Perceived Financial Vulnerability: A Normative Study

**DOI:** 10.1093/geroni/igaa039

**Published:** 2020-09-07

**Authors:** Peter A Lichtenberg, Daniel Paulson, S Duke Han

**Affiliations:** 1 Institute of Gerontology and Department of Psychology, Wayne State University, Detroit, Michigan; 2 Department of Psychology, University of Central Florida, Orlando; 3 Departments of Family Medicine, Neurology, and Psychology and School of Gerontology Keck School of Medicine, University of Southern California, Los Angeles

**Keywords:** Financial strain, Mental health, Wealth

## Abstract

**Background and Objectives:**

Age-associated financial vulnerability was introduced because it was increasingly recognized that cognitively intact older adults experienced changes that rendered them financially vulnerable. In this study, we attempt to apply the construct of Age-Associated Financial Vulnerability to a measure of Perceived Financial Vulnerability and whether this perceived vulnerability is predicted by risk factors from the 4 categorical domains used to define Age-Associated Financial Vulnerability’s impact.

**Research Design and Methods:**

This study was part of the Health and Retirement Study (HRS) survey in 2018. The survey contained 7 experimental module items of Perceived Financial Vulnerability. One thousand three hundred fourteen participants completed the Perceived Financial Vulnerability measure. The sample was drawn from Waves 13 and 14 of the HRS (2016 and 2018, respectively). The measurement of Perceived Financial Vulnerability was developed on the basis of 7 questions assessing financial awareness and psychological vulnerability items regarding personal finance that were included in the 2018 HRS data collection. Predictors included measures of cognition, function/health, depression, and wealth. Predictor measures from 2016 were regressed on 2018 Perceived Financial Vulnerability scores.

**Results:**

Six items of Perceived Financial Vulnerability had psychometric properties acceptable for a new measure. Responses revealed variability in Perceived Financial Vulnerability. Overall, 18% of variance was accounted for and measures from cognition, depression, assets, and functional abilities were all unique and significant predictors.

**Discussion and Implications:**

This study represents both a conceptual and empirical contribution to our understanding of older adult’s perceptions of financial vulnerability. The high levels of Perceived Financial Vulnerability found in this normative sample underscore the importance of context in understanding people’s economic behaviors. For instance, more than one half of the sample indicated that they wished they had someone to talk to about their finances. This desire to talk with others is normative and yet often underappreciated.

**Translational Significance:** The linkages between personal finances and health are becoming better understood as integral to well-being in older age. Perceived Financial Vulnerability, to date, understudied, is a useful brief assessment and can become a target for intervention. This study provides a set of questions to measure this aspect of personal finance.

The term “age-associated financial vulnerability” (Age-Associated Financial Vulnerability; Lachs & Han, 2015) was introduced because it was increasingly recognized that cognitively intact older adults experience changes that render them financially vulnerable. Age-Associated Financial Vulnerability is defined as a pattern of financial behavior that places an older adult at substantial risk for a considerable loss of resources due to financial decision making that is inconsistent with the financial decision-making patterns established earlier in the person’s life. Risk factors that contribute to Age-Associated Financial Vulnerability can be categorized into four major domains: cognitive/emotional, medical and functional, psychosocial, and environmental/societal. Understanding financial vulnerability in older adults—both susceptibility to financial exploitation (FE) and to deficits in informed decision making (i.e., financial decision-making capacity)—is becoming steadily more important, given recent increases in both the experience of financial victimization in the older population and its reporting. For instance, according to the Consumer Financial Protection Bureau (2019), in only 4 years (2013–2017) deposit institutions and financial services businesses filed four times as many Suspicious Activity Reports. Almost 70% of these reports were for individuals older than 60 and 33% for those older than 80. In this study, we apply the Age-Associated Financial Vulnerability construct to a measure of Perceived Financial Vulnerability and examine whether this perceived vulnerability is predicted by risk factors from the four categorical domains.

## FE as One Outcome of Financial Vulnerability

FE, one outcome of financial vulnerability, has been studied through population-based surveys in several samples of nondemented older adults. Beginning in 2010, the prevalence of FE and its correlates was examined in population-based samples. Acierno et al. (2010) reported that 5.2% of all older adults in their sample had experienced FE during the previous year, and 60% of such instances consisted of family members’ misappropriation of money. Laumann et al. (2008) found that 3.5% of their sample had been victims of FE during the previous year. Younger older adults (aged 55–65) and African Americans were more likely to report FE, and participants with a romantic partner were less likely. Beach et al. (2010) found that 3.5% of their sample reported experiencing FE during the previous 6 months, and almost 10% had at some point since turning 60. In their sample, African Americans were again more likely to report FE. The authors found that depression and impaired activities of daily life (ADLs) were additional correlates of FE. These studies demonstrate that the prevalence of FE in older adults is high and that it is a multidimensional problem with health, sociodemographic, and psychological correlates.

The FE literature has sought to identify the risk factors that render older adults more vulnerable to victimization. These include younger-old age (Acieno et al., 2010; Boyle et al., 2012; Garre-Olmo et al., 2009), poor physical health (Wood et al., 2016), and less fulfillment of social needs or limited social support networks (Choi & Mayer, 2000; Lichtenberg et al., 2013). Other risk factors include low performance on measures of financial skills and numeracy (Wood et al., 2014), less financial satisfaction (Lichtenberg et al., 2013), lower levels of education (Boyle et al., 2012), and lower literacy (James et al., 2014). The Rush Alzheimer’s Disease Center (Boyle et al., 2012, 2013; Han et al., 2016) has contributed greatly to the literature on financial decision making and scam susceptibility, which is a form of FE. The studies cited above not only link declines in financial decision making to reduced cognition—even without dementia—but also link brain regions and findings on decision making to scam susceptibility.

Burnes et al. (2017) conducted a meta-analysis to measure the prevalence of a specific form of FE—fraud—and demonstrated that there is great variability in how fraud is measured and how that variability is related to prevalence rates. Overall, a prevalence rate of 5.6% fraud victimization per year was reported. Shao et al. (2019) focused their review on what renders older adults vulnerable to fraud. While there is considerable debate about whether older adults are more susceptible to fraud than other age groups, Shao et al. focused on six types of phenomena that were related to fraud in some studies or conceptualizations: cognitive functioning and cognitive decline (James et al., 2014); emotional regulation and the positivity effect—the tendency of older adults to have reduced arousal response to negative circumstances surrounding a financial decision and to have positive expectations around decision making (Spreng et al., 2016); the interplay between personal competencies and the environment (Pinkser et al., 2009); social isolation (Alves & Wilson, 2008); risk taking (Samanez-Larkin et al., 2007); and psychological vulnerability (Lichtenberg et al., 2016).

The risk factors for FE and fraud are consistent with those outlined for Age-Associated Financial Vulnerability. Cognitive, functional, psychological, and financial variables all are noted risk factors for FE. FE can be thought of as one outcome of Age-Associated Financial Vulnerability. As a construct, however, Age-Associated Financial Vulnerability is not meant to be identical to FE. An older adult’s perception of various aspects of finances and financial transactions may represent one way to measure financial vulnerability. Financial vulnerability can be a useful measure not only for the risk of exploitation, but also to better understand the nature of the perceived challenges older adults face regarding finances. What is the normative experience of Perceived Financial Vulnerability for older adults?

The purpose of this study was to examine an experimental module consisting of questions designed to measure Perceived Financial Vulnerability on the 2018 Health and Retirement Study (HRS). Specifically, we sought to discover how prevalent Perceived Financial Vulnerability is in a population-based sample, and whether a set of questions about aspects of financial vulnerability constitute an internally consistent measure. In addition, we aimed to determine whether Age-Associated Financial Vulnerability predictors from the domains of cognition, health, psychological factors, and wealth, along with demographic variables from the 2016 survey, were significant predictors of the 2018 Perceived Financial Vulnerability measure scores. Finally, preliminary construct validity was assessed using a reduced subsample by examining the relationship between subjective financial knowledge, history of economics coursework, distrust of financial professionals, and fear of financial insufficiency.

## Method

### Sample

**Figure 1.**
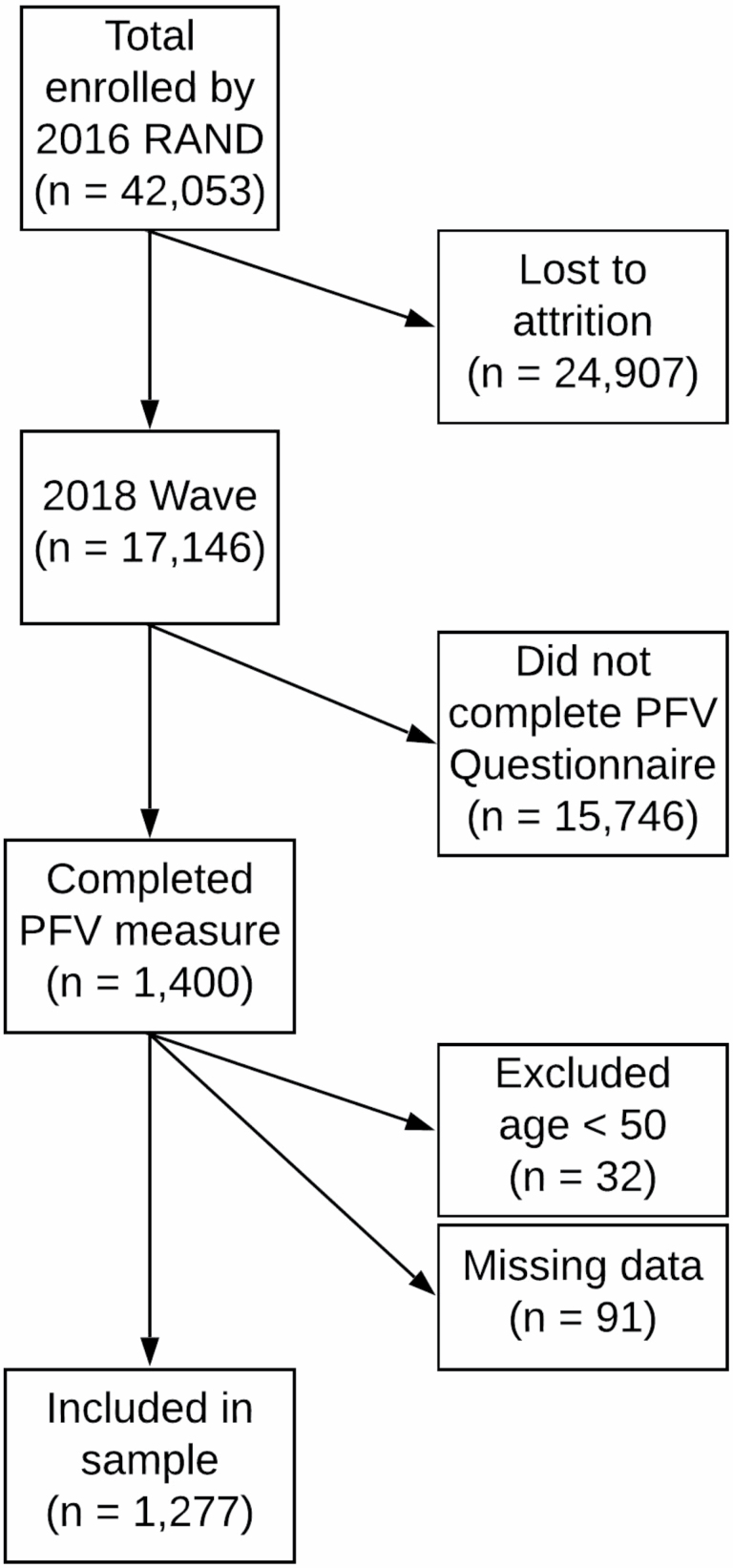
CONSORT diagram.

The HRS is a longitudinal panel study funded by the National Institutes of Health, with data collection centralized through the University of Michigan. Information on HRS design and collection methods can be found in published reports (Heeringa & Conner, 1995). The first HRS wave was enrolled in 1992 and followed up at 2-year intervals. At the time of this writing, the overall HRS data set includes 42,053 respondents overall and just more than 17,000 respondents in the 2018 data collection, of whom 1,314 were randomly selected and completed the Perceived Financial Vulnerability measure and were included in this study. Of those, overlapping patterns of missing data reduced the sample to 1,277 participants (see CONSORT diagram in Figure 1). The sample was drawn from Waves 13 and 14 of the HRS (2016 and 2018, respectively).

### Measures

All variables were gathered from the 2016 wave, with the exception of the Perceived Financial Vulnerability measure, which was gathered in the 2018 wave.

Demographic questionnaire items were used to assess age, gender, marital status, years of education, and Hispanic ethnicity. Total household earnings and total household assets were calculated on the basis of questions about various sources of income and wealth, respectively. Both variables have skewed distributions, which were addressed by stratifying them into pentiles.

Depressive symptoms were assessed using the eight-item Center for Epidemiological Studies-Depression (CES-D) measure, which is based on the original longer index of the same name (Radloff, 1977). Participants answered “yes” or “no” to statements about their feelings “much of the time” in the past week. Two items were positively worded (“enjoyed life” and “was happy”) and six were negatively worded (e.g., “felt depressed,” “felt that everything I did was an effort”). Scores ranged on a scale from 0 to 8, with higher scores suggesting higher levels of depression. The measure has a high Cronbach’s alpha of 0.77 (Steffick, 2000) and is widely used in studies of late-life depression (Beekman et al., 1997).

Morbidity was assessed on the basis of self-reported diagnosis of common medical conditions: hypertension, diabetes, cancer, lung disease, heart disease, stroke, psychiatric problems, and arthritis. Scores ranged from 0 to 8.

ADLs were assessed on the basis of subjective reports of needing assistance with bathing, eating, dressing, walking across a room, and getting in or out of bed. Scores ranged from 0 to 5. Instrumental activities of daily living (IADLs) were measured by identifying which of the following the respondent required assistance with: using a telephone, taking medication, and handling money (Wallace & Herzog, 1995). Scores ranged from 0 to 3.

The 35-point standardized cognitive status measure was based on the Telephone Interview for Cognitive Status (Brandt et al., 1988), which includes indices of orientation, concentration, short-term memory, working memory (serial sevens), praxis, and language. This index has a Cronbach’s alpha of 0.69 (Herzog & Wallace, 1997), high test–retest reliability, and adequate sensitivity to cognitive impairment (Brandt et al., 1988; Desmond et al., 1994; Järvenpää et al., 2002; Welsh et al., 1993). Higher scores on the Mini-Mental State Examination and serial sevens indicate better cognitive functioning.

Distrust of financial advisors was assessed with a single question, “How much do you trust bankers or other professional financial advisors to provide you with useful information about your money decisions? Would you say that you trust them very much (1), somewhat (2), not very much (3), or not at all (4)?” Subjective financial knowledge was assessed using a single question with a 7-point Likert-type response scale ranging from (1) very low to (7) very high. Formal economics education was assessed on the basis of participants’ response to the dichotomously scored question: “During your school education, that is high school, college or graduate school, did you take any courses in economics or finance?”

## Development of the Perceived Financial Vulnerability

The Perceived Financial Vulnerability measure was derived from the contextual items of the Lichtenberg Financial Decision Rating Scale (Lichtenberg et al., 2015), a 56-item scale. Lichtenberg et al. (2015) proposed a new conceptual model to understand financial decision making and utilize in financial capacity assessments. The model incorporates contextual variables with the Appelabaum and Grisso (1988) decision-making model. Appelbaum and Grisso (1988) elaborated on the decision-making model (what we termed as the intellectual factors) involved in capacity assessment: choice, understanding, appreciation, and reasoning. These kernel intellectual factors have been reiterated as fundamental aspects of decisional abilities (ABA/APA, 2008). Contextual subscales included Financial Awareness, Psychological Vulnerability, and Susceptibility. The contextual factors are self-reported items and encompass personal finance areas such as financial strain, financial self-efficacy, financial satisfaction, anxiety or depression regarding finances, the presence of or loss of a confidante with whom finances were discussed, relationship strain due to finances, and conflicts about how money is spent.

The Perceived Financial Vulnerability was derived from the 34 contextual items of the Lichtenberg scale. We applied to the HRS to have an experimental module used in the 2018 survey. In consultation with the HRS, seven items were chosen to be included—these were the basis for the Perceived Financial Vulnerability. The measurement of Perceived Financial Vulnerability was developed based on seven items that assess financial awareness and psychological vulnerability regarding personal finances, which were included in the 2018 HRS data collection. Question 4 was dichotomously worded with very little variability and therefore excluded. The final score on this measure was the sum of six questions answered using a 3-point Likert-type scale. Scores could range from 6 to 18. Item wording is presented in Table 2. Psychometric information is reported below.

### Statistical Methods

Internal consistency of this scale was assessed on the basis of inter-item correlations, Cronbach’s alpha, and exploratory factor analysis. Validity was assessed by examining hypothesized correlates with demographic, medical, cognitive, and psychological variables (listed above). Finally, a hierarchical regression procedure was employed to characterize the relationship between Perceived Financial Vulnerability and other variables of interest in a multivariate fashion. This strategy is a common approach, particularly in social sciences research, for analyses in which predictor variables are correlated (Pedhazur, 1997). Hierarchical regression is also useful when theoretical considerations guide important questions about the degree to which primary variables of interest account for variance in the criterion over or above variance accounted for by control or contextual factors. The hierarchical linear regression strategy employed in this study controlled for the influence of demographic variables explained variance in Perceived Financial Vulnerability (Step 1) before assessing the relative contribution of wealth (Step 2). Step 3 sought to determine whether medical, psychological, and cognitive accounted for variance in Perceived Financial Vulnerability over and above that associated with demographic characteristics and wealth. Findings are reported using unstandardized regression weights. Finally, correlation analyses and independent-samples *t* tests were employed to examine how Perceived Financial Vulnerability associates with financial knowledge, distrust of financial professionals, and fear of financial insufficiency.

## Results

The final sample of 1,314 participants was predominantly female (58.9%), White/Caucasian (68.3%), and partnered (63.9%). Participants ranged in age from 50 to 101 years. Demographic characteristics are summarized in Table 1. Table 2 presents the frequencies for the seven financial vulnerability items administered. As noted, Question 4 was excluded from the calculation of Perceived Financial Vulnerability. Perceived Financial Vulnerability scores had a mean of 8.8 and a standard deviation of 2.1, a median score of 8. A score of 7 captured the lowest quartile, a score of 8 the second, a score of 10 the third, and 17 the fourth. Financial vulnerability items indicated that many older adults in this population have experienced various aspects of vulnerability. Nearly 65% of the sample reported anxiety about a financial decision sometimes or often. In other responses, more than 50% of the sample wished they had someone to talk to about finances, and nearly 30% worried that someone would take away their financial freedom. Twenty-five percent of the sample lacked confidence in their financial decision making, and nearly one third reported being treated with less respect than others during a financial transaction.

**Table 1.** Descriptive Statistics

	Range	Mean (*SD*) or median (SIR)	% of Sample
Age	50–101	67.5 (10.7)	
Years of education	0–17	12.9 (3.2)	
CES-D	0–8	1.5 (2.0)	
Morbidity	0–8	2.2 (1.6)	
ADLs	0–5	0.3 (0.8)	
IADLs	0–3	0.1 (0.4)	
Serial sevens	0–5	3.5 (1.7)	
Total cognition	6–35	23.5 (4.3)	
Household income	$0–$1.5M	$46,356 ($35,518)	
Household assets	−$291,500 to $11.7M	$132,400 ($202,913)	
Female			58.4
Race			
White			68.6
Black			20.6
Other			10.2
Hispanic			14.9
Partnered			63.9

*Note:* ADLs = activities of daily living; CES-D = Center for Epidemiological Studies-Depression; IADLs = instrumental activities of daily living; SIR = semi interquartile mean.

**Table 2.** Question Response Frequencies (*N* = 1,314)

Question text	Response options	Frequencies
How often do you feel anxious about your day-to-day financial decisions or transactions?	*Never*	35.4%
	*Sometimes*	48.9%
	*Often*	15.8%
How often do you wish that you had someone to talk to about your financial decisions, transactions, or plans?	*Never*	45.2%
	*Sometimes*	45.0%
	*Often*	9.8%
How worried are you that someone will take away your financial freedom?	*Not at all worried*	67.9%
	*Somewhat worried*	25.3%
	*Very worried*	6.8%
In the past 6 months, have you had any conflicts with anyone (other than your spouse/partner) about the way you spend money or to whom you give money?	*Yes*	96.4%
	*No*	3.5%
How confident are you in making big financial decisions?	*Confident*	73.8%
	*Unsure*	19.9%
	*Not confident*	6.2%
When it comes to making financial decisions and transactions, how often are you treated with less courtesy and respect than other people?	*Never*	70.2%
	*Sometimes*	23.4%
	*Often*	6.3%
How often has someone talked you into a decision to spend or donate money that you did not initially want to do?	*Never*	77.9%
	*Sometimes*	20.0%
	*Often*	2.1%

Inter-item correlations, reported in Table 3, ranged from 0.10 to 0.40. The remaining six items demonstrated marginal internal consistency (Cronbach’s α = 0.59), which would not be appreciably improved by the exclusion of any of the remaining six items. The Kaiser-Meyer-Oklin value of 0.721 indicates an acceptable proportion of common variance. Results of a principal component analysis showed that a single factor best accounts for variance in these items (*Eigenvalue* = 2.0). Factor loadings, listed in Table 3, range from 0.381 to 0.708.

**Table 3.** Inter-item Correlations and Scale Attributes

	Item 1	Item 2	Item 3	Item 5	Item 6	Item 7
Item 2	0.40	1				
Item 3	0.32	0.28	1			
Item 5	0.15	0.12	0.14	1		
Item 6	0.19	0.16	0.16	0.12	1	
Item 7	0.16	0.18	0.22	0.10	0.16	1
Cronbach’s α ^a^ if deleted	0.49	0.51	0.52	0.59	0.57	0.57
Component matrix	0.71	0.67	0.65	0.38	0.48	0.49
Factor loading	0.71	0.67	0.65	0.38	0.48	0.49

*Note:* All items correlate (*p* < .05).

^a^Cronbach’s α for the scale = 0.59.

Univariate correlations, given in Table 4, indicate that total Perceived Financial Vulnerability scores were associated with each of the four categorical domain variables of Age-Associated Financial Vulnerability. In addition, demographic factors were significantly related to Perceived Financial Vulnerability scores. Significant demographic variables related to Perceived Financial Vulnerability included being unpartnered (*r* = −0.08) and non-White (*r* = −0.14). Perceived Financial Vulnerability scores were negatively correlated with age (*r* = −0.16), education (*r* = −0.06), total household income (*r* = −0.21), and overall assets (*r* = −0.29). With regard to cognition, Perceived Financial Vulnerability scores were significantly correlated with serial sevens (*r* = −0.17) and total cognition based on the 35-point measure (*r* = −0.16). Perceived Financial Vulnerability was positively correlated with the psychological measure of depression (CES-D score, *r* = 0.34) and the medical measure of chronic conditions (morbidity, *r* = 0.14). Perceived Financial Vulnerability scores were also significantly correlated with deficits in ADLs (*r* = 0.19) and IADLs (*r* = 0.19; *p* < .05 for all). Scores on the Perceived Financial Vulnerability assessment were not significantly associated with gender (*r* = 0.03, *p* > .05) or Hispanic ethnic identity (*r* = 0.05, *p* > .05). It should be noted that the 2016 data collection is missing data required to compute the 35-point cognitive status score on 425 respondents who completed the Perceived Financial Vulnerability measure in 2018. However, serial sevens was complete for almost all participants. As a result, the serial sevens, which is often interpreted as an index of working memory, was used in lieu of the longer 35-point total cognition assessment in the following multivariate analysis.

**Table 4.** Correlation Matrix

	1	2	3	4	5	6	7	8	9	10	11	12	13	14
1. Perceived Financial Vulnerability	1													
2. Gender	0.03	1												
3. Partnered	−0.08*	−0.20*	1											
4. Age	−0.16*	0.01	−0.14*	1										
5. Education	−0.06*	−0.08*	0.09*	−0.06*	1									
6. Minority	−0.14*	−0.06*	0.15*	0.24*	0.18*	1								
7. Hispanic	0.05	0.07*	0.02	−0.14*	−0.37*	−0.13*	1							
8. Earnings	−0.21*	−0.13*	0.45*	−0.14*	0.44*	0.23*	−0.21*	1						
9. Assets	−0.29*	−0.08*	0.30*	0.19*	0.34*	0.31*	−0.18*	0.54*	1					
10. CES-D	0.34*	0.13*	−0.23*	−0.06*	−0.17*	−0.11*	0.09*	−0.31*	−0.28*	1				
11. Morbidity	0.14*	0.02	−0.12*	0.31*	−0.10*	0.02	−0.09*	−0.24*	−0.14*	0.29*	1			
12. ADLs	0.19*	0.06*	−0.14*	0.04	−0.08*	−0.08*	0.05	−0.21*	−0.19*	0.36*	0.31*	1		
13. IADLs	0.19*	0.04	−0.10*	0.01	−0.15*	−0.06*	0.16*	−0.18*	−0.18*	0.25*	0.19*	0.39*	1	
14. Serial Sevens	−0.17*	−0.12*	0.13*	−0.06*	0.38*	0.21*	−0.16*	0.35*	0.29*	−0.23*	−0.14*	−0.15*	−0.21*	1
15. Total Cognition	−0.16*	0.04	0.10*	−0.18*	0.42*	0.16*	−0.10*	0.35*	0.27*	−0.15*	−0.18*	−0.17*	−0.13*	0.66*

*Notes:* ADLs = activities of daily living; CES-D = Center for Epidemiological Studies-Depression; IADLs = instrumental activities of daily living. Gender is coded (0 = male, 1 = female). Partnership status is coded (0 = not partnered, 1 = partnered). Minority status is coded (0 = non-White, 1 = White).

*p < .05.

To examine which variables from the 2016 wave predicted 2018 Perceived Financial Vulnerability scores, we used a stepwise multivariate analysis and found that many of these results remained as reported in Table 5. In the first step, using only demographic measures, the Perceived Financial Vulnerability score was predicted by being unpartnered (*B* = −0.35, *SE* = 0.12, *p* < .05), younger (*B* = −0.03, *SE* = 0.01, *p* < .05), and having a non-White racial identity (*B* = −0.37, *SE* = 0.13, *p* < .05). In combination, these variables accounted for 4.5% of the variance in Perceived Financial Vulnerability scores. In the second step, the addition of household wealth measures significantly increased the percentage of variance accounted for to 9.9% (*p* < .05). In this step, Perceived Financial Vulnerability continued to be associated with age (*B* = −0.02, *SE* = 0.01, *p* < .05), but partnership status and racial status were no longer significant predictors (*p* > .05). Notably, household earnings (*B* = −0.22, *SE* = 0.06, *p* < .05) and household assets (*B* = −0.30, *SE* = 0.05, *p* < .05) were found to be significant predictors of Perceived Financial Vulnerability. The third step also significantly increased the variance accounted for to 18.5% (*p* < .05) with the addition of cognition, depression, medical conditions, and functional abilities. The Perceived Financial Vulnerability score continued to be associated with younger age (*B* = −0.03, *SE* = 0.01, *p* < .05). Years of education (*B* = 0.06, *SE* = 0.02, *p* < .05) emerged as a significant predictor in the third step, and the direction of the relationship between partnership status and Perceived Financial Vulnerability score was reversed (*B* = 0.25, *SE* = 0.13, *p* < .05). This suggests that after accounting for other demographic, financial, cognitive, and medical, psychological, and functional factors, being partnered was associated with higher financial vulnerability. Additionally, Perceived Financial Vulnerability performance was positively associated with IADL disability (*B* = 0.45, *SE* = 0.16, *p* < .05), negatively associated with performance on the serial sevens task (*B* = 0.09, *SE* = 0.04, *p* < .05), and positively associated with endorsement of depressive symptoms on the CES-D (*B* = 0.25, *SE* = 0.03, *p* < .05).

**Table 5.** Results of Multiple Regression Analysis in Which 2016 Correlates Predict 2018 Perceived Financial Vulnerability Score (*N* = 1,277)

	Step 1		Step 2		Step 3	
	*B*	*SE*	*B*	*SE*	*B*	*SE*
Constant	11.7*	0.50	11.51*	0.50	10.43*	0.49
Gender	0.03	0.12	0.01	0.11	−0.08	0.11
Partnered	−0.35*	0.12	0.14	0.13	0.25*	0.13
Age	−0.03*	0.01	−0.02*	0.01	−0.03*	0.01
Years of education	−0.04	0.02	0.03	0.02	0.06*	0.02
Minority/White	−0.37*	0.13	−0.14	0.13	−0.12	0.12
Hispanic	0.00	0.18	−0.12	0.17	−0.15	0.17
Earnings			−0.22*	0.06	−0.11*	0.05
Household assets			−0.30*	0.05	−0.22*	0.05
CES-D					0.25*	0.03
Medical burden					0.07	0.04
ADL disability					0.05	0.07
IADL disability					0.45*	0.16
Serial sevens					−0.09*	0.04
% Variance accounted for	4.1*		9.9*		18.5*	

*Notes:* ADL = activities of daily living; CES-D = Center for Epidemiological Studies-Depression; IADL = instrumental activities of daily living. Gender is coded (0 = male, 1 = female). Partnership status is coded (0 = not partnered, 1 = partnered). Minority status is coded (0 = non-White, 1 = White).

*p < .05.

Some final analyses examined Perceived Financial Vulnerability construct validity in a reduced subsample (*N* = 119–125). Respondent Perceived Financial Vulnerability had a nonsignificant, negative relationship with subjective financial knowledge in the prior wave (*r* = −0.18, *p* = .052), though respondents with a history of formal economics education reported less Perceived Financial Vulnerability than did those who denied having completed coursework on this subject (*t*_(*df* = 120)_ = −2.34, *p* = .02, *d* = 0.43). Respondent distrust of financial advisors (*r* = 0.22, *p* = .01) and subjective fear of financial insufficiency (*t*_(*df*=118)_ = −4.74, *p* < .001, *d* = 0.87) were both positively associated with Perceived Financial Vulnerability.

## Discussion

Empirical evidence supports the idea that perceived financial vulnerability (Perceived Financial Vulnerability), consistent with the construct of age-associated financial vulnerability (Age-Associated Financial Vulnerability), puts older adults at more risk of losing considerable resources (Lichtenberg et al., 2020). This study examines other aspects of how Perceived Financial Vulnerability relates to the construct of Age-Associated Financial Vulnerability in a population-based sample of adults older than 50. Specifically, we identified four categories of variables that are conceptually related to Age-Associated Financial Vulnerability: cognitive, medical/functional, psychological, and environmental. Our results demonstrate that even after controlling for demographic predictors of Perceived Financial Vulnerability, predictors from each of the four categories were significantly related to Perceived Financial Vulnerability. Wealth and income cumulatively accounted for 5.8% of variance over and above demographic correlates. Medical, psychosocial, and cognitive predictors accounted for an additional 8.6% of variance in the Perceived Financial Vulnerability score. These included measures of serial sevens (cognition), depressive symptoms (psychological), IADLs (functional), and household wealth and income (environmental). It is interesting that Perceived Financial Vulnerability was strongly associated with the fear of financial insufficiency and distrust of financial professionals. In combination with findings that formal schooling in economics is associated with reduced Perceived Financial Vulnerability, findings suggest interesting avenues for intervention that may involve life-span training in financial services and financial preparedness, with targeted fraud resistance training for older adults. While Perceived Financial Vulnerability maps well on the construct of financial vulnerability overall, the issue of how our findings relate to age-associated financial vulnerability is more complex.

Some may interpret Age-Associated Financial Vulnerability as supporting the notion that as a person ages, so will their Perceived Financial Vulnerability. This was not supported in this study: Even when accounting for all other predictors, age was inversely related to Perceived Financial Vulnerability—that is, younger-old individuals reported the most Perceived Financial Vulnerability. Age was correlated with decreased wealth and more chronic conditions, but negatively correlated with depressive symptoms. The finding that younger-old adults are more vulnerable has also been reported in studies on FE (Acierno et al., 2010; Lichtenberg et al., 2013).

However, Age-Associated Financial Vulnerability can be thought of more broadly, as referring to vulnerability older than the age of 50. Age and cohort effects are hard to untangle in this type of study. The younger-old in our cohort may have experienced more job layoffs and/or, for example, more insecurity about retirement funds given the increasing loss of pensions. In this case, vulnerability may be more related to cohort and not simply age. Further complicating matters is the difference between Perceived Financial Vulnerability and actual financial vulnerability; there may be a disconnect between the two that is linked to age, education, literacy, or cognition. More work is needed to explore the associations between perceived and actual financial vulnerability and their potentially differential effects on health and well-being.

The high levels of Perceived Financial Vulnerability found in this normative sample underscore the importance of context in understanding people’s economic behaviors and vulnerabilities. For instance, more than one half of the sample reported wishing they had someone to talk to about their finances. This desire to talk with others is normative, but one’s choice of confidant may increase or decrease the risk of negative financial impacts; abuse of trust (Conrad et al., 2010) is often a primary component of older adults’ FE. Notably, scammers are well aware that many older adults are lonely and routinely employ tactics to decrease a lonely person’s anxiety in the short term to gain access to the person’s money in the long term.

Alternatively, our findings suggest that having a trustworthy confidant mitigates perceived vulnerability and protects financial well-being overall. DeLiema (2018) investigated routine activity theory as a context for fraud susceptibility and found that isolation and a lack of trustworthy friends or family best distinguished those who had been defrauded from those who had not. Routine activity theory requires the convergence of three factors: an offender, a target, and the absence of others to protect the target. A study by Resig and Holtfreter (2013) found that when the target has low self-control they were more likely to be a victim of mail fraud. Lichtenberg et al. (2013) found that a combination of high depression and low sense of status best predicted who was most vulnerable to fraud victimization. In these normative data, we found that 30% of the sample perceived themselves as being treated with less courtesy and respect (i.e., status) than other people during financial transactions.

The primary limitation of this study is that infrequent endorsement of financial fraud by any mechanism prevented explicit identification of a cut point that may guide decision making in research and clinical settings. The second shortcoming of this study is the finding of a modest Cronbach’s alpha. This finding suggests suboptimal internal consistency, though this finding is somewhat ambiguous given the relative brevity of the measure. The diverse range of correlates identified by this study suggests multiple avenues to Perceived Financial Vulnerability, and future research may further investigate how diversity among respondents relates to this and other psychometric properties of the Perceived Financial Vulnerability measure. The use of 2016 Perceived Financial Vulnerability correlates provides both benefits and shortcomings. The use of retrospective correlates reflects the conceptualization of Perceived Financial Vulnerability as a “downstream” construct reflecting the joint influence of various risk factors. The use of 2016 HRS correlates makes use of valuable data collected only during the prior wave (financial knowledge, fear of financial insufficiency, in particular). By contrast, it would be very informative to better understand how Perceived Financial Vulnerability cross-sectionally relates to demographic, health, and other correlates. The Perceived Financial Vulnerability data were derived from early-release 2018 HRS data, and the computation of several key correlates using 2018 data was impracticable or impossible at the time of this writing. Future research should address the cross-sectional overlap between these variables as the finalized 2018 data become available.

This study represents an early step toward understanding Perceived Financial Vulnerability in older adults and its relationship to the construct of Age-Associated Financial Vulnerability. A strength of the study is that the predictors of Perceived Financial Vulnerability were measured 2 years before the vulnerability measure. A weakness of the study is that the 2018 wave of the HRS was the first time financial vulnerability was measured and thus cannot be explored as a predictor variable for health conditions. However, the four categories implicated by Age-Associated Financial Vulnerability were found to be significant predictors of perceived vulnerability. This study thus represents both a conceptual and empirical contribution to our understanding of older adults’ perceptions of financial vulnerability.
